# A mechanistic model explains variation in larval tick questing phenology along an elevation gradient

**DOI:** 10.1098/rsos.250130

**Published:** 2025-04-30

**Authors:** David Allen

**Affiliations:** ^1^ Department of Biology, Middlebury College, Middlebury, VT, USA

**Keywords:** phenology, elevation, *Ixodes scapularis*, demography, mechanistic model

## Abstract

Many tick-borne pathogens are maintained in enzootic cycles passing from nymphs of one tick cohort to larvae of the next via vertebrate hosts. As such, the phenology of larval and nymphal host-seeking, questing, partially determines pathogen persistence. Across the range of the blacklegged tick (*Ixodes scapularis*), the timing of larval phenology varies due to differences in climate and local adaptation in the timing of temperature-independent diapause. In this study, an elevation gradient was used to isolate climate as temperature varies with elevation over small geographic scales where local adaptation should be absent. The ability of a mechanistic, temperature-driven, literature-parametrized model to explain variation in larval *I. scapularis* phenology was tested. Over 7 years, *I. scapularis* ticks were collected using drag-cloth sampling along a > 500 m elevation gradient in western Vermont, USA. At low elevation, more larval ticks quested in late summer, while at high elevation, more quested in early summer. The literature-parametrized model reproduced these differences better than competing models. This validated model provides an explicit, mechanistic connection between temperature and larval phenology, a key determinant of tick-borne disease persistence.

## 1. Introduction

Ticks are vectors of important wildlife, livestock and human pathogens [1]. These pathogens exist in enzootic cycles between tick vectors and vertebrate reservoirs. Although some tick-borne pathogens are maintained through vertical transmission [2,3], most tick-borne pathogens of human concern are maintained through horizontal transmission. For these pathogens, the predominant transmission pathway consists of transmission from an infected nymph to a vertebrate host during nymphal feeding, followed by transmission from that host to a larval tick during larval feeding [4,5]. Larval ticks molt to be infected nymphs completing the pathway.

Therefore, enzootic persistence requires the pathogen to stay infectious in the vertebrate host between periods of nymphal and larval feeding. Thus, the relative timing of feeding for these tick stages and duration of pathogen infectiousness in the vertebrate host are key to enzootic persistence [6–8]. For example, tick-borne encephalitis virus (TBEv) is only found in areas of Europe with synchronous larval and nymphal *Ixodes ricinus* feeding [9]. TBEv persists for a short period of time in vertebrate hosts, so in areas of Europe where nymphal and larval feeding are asynchronous, the virus cannot persist [9]. Different strains of *Borrelia burgdorferi*, the Lyme disease agent, stay infectious in the vertebrate hosts for different amounts of time [10,11]. As such, asynchronous larval and nymphal phenology could select for long-duration infectious *B. burgdorferi* strains. And indeed, regions of the United States with asynchronous *Ixodes scapularis* larval and nymphal phenology have a higher fraction of *B. burgdorferi* strains causing longer duration infections [12,13]. These strains are more virulent in humans [14], so tick questing phenology can ultimately have human disease risk consequences.

This connection between tick phenology and tick-borne pathogen persistence means it is important to understand the drivers of tick phenology. Across the range of a tick species, there can be variation in questing phenology. For example, larval *I. scapularis* typically quest in late summer in the northeastern United States and early summer in the upper Midwest [12,13,15]. In both regions, nymphal ticks generally quest from late spring to early summer. *Ixodes ricinus* larval phenology also varies across its range [9]. Attempts have been made to understand how climate drives larval phenology in these species [15,16]. Gatewood *et al*. [12] suggested late-summer larval activity is determined by the rate of autumnal cooling. Slower autumnal cooling in the oceanic climate of the eastern United States allowed larval ticks to quest into late summer, while faster cooling in the continental climate of the midwestern United States caused larvae to overwinter and quest early in the following summer [12]. This idea was supported by Levi *et al*. [17] who found that across 19 years in a single location, the proportion of late-summer questing larvae was positively correlated with the autumn temperature.

Although these phenomenological frameworks provide some insight into what drives variation in larval phenology, a mechanistic understanding is needed. Such a framework would explicitly account for how temperature drives the individual lifecycle processes (e.g. oviposition, eclosion and questing) which ultimately determine questing phenology. Ogden *et al*. [15] provided such a mechanistic model and tested whether temperature differences could explain the variation in larval phenology of *I. scapularis* across the eastern United States. They found that no single temperature-driven model could explain regional patterns. Instead, they found regional differences in the fraction of larvae which enter temperature-independent diapause after the summer solstice, likely due to local adaptation, which explained regional differences in phenology. As such, they were not able to test whether a single model, based solely on temperature, could explain the differences in larval phenology.

In this study, the ability of a mechanistic, temperature-driven model to explain differences in larval phenology along an elevation gradient was tested. Elevation gradients provide large climate variation over short geographic distances, which should minimize the role of local adaptation. So here, larvae should have a similar pattern of diapause after the summer solstice while experiencing different climates. Larval *I. scapularis* questing phenology was measured along a 500 m elevation gradient. A temperature-dependent, mechanistic model of larval tick phenology was developed and parametrized with values from the literature. The performance of this model was compared against competing models.

## 2. Methods

### 2.1. Study organism

*Ixodes scapularis* is a hard tick with three life-active stages: larva, nymph and adult. At each stage, they use a ‘sit and wait’ strategy, called questing, to search for a potential host in the leaf litter or on understory vegetation. They take a blood meal from that host, fall off and molt to the next life stage. They feed from a wide diversity of vertebrate hosts [18] and are vectors of many pathogens, including the Lyme disease agent [19].

### 2.2. Tick sampling and leaf litter temperature

**Figure 1.**
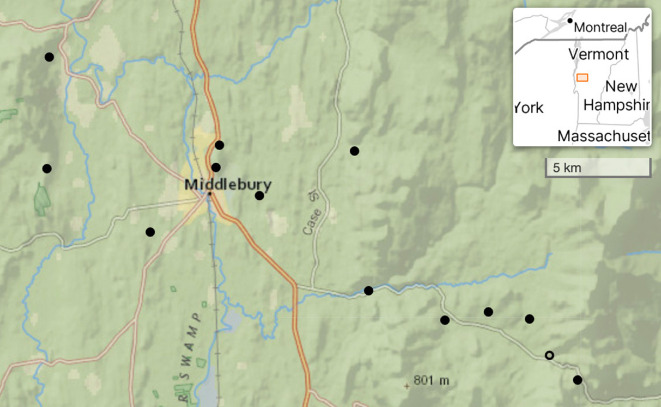
A map of the 13 tick sampling sites. Larvae were found at sites indicated with filled circles and were not found at the one site indicated with an open circle. Base map courtesy of ERSI. Sites around Middlebury and to the west are in the Champlain Valley. Those to the east are in the Green Mountains.

Ticks were sampled at 13 sites along an elevation gradient (126–683 m) in the Champlain Valley and western slopes of the Green Mountains in Vermont, USA (figure 1) with drag-cloth sampling. This is a standard method to sample for questing ticks [20]. At each site, two or three 200 m^2^ sampling plots were established. All plots were located within closed canopy, predominately deciduous forests (see [21] for more details). Sites were sampled every two to four weeks from 1 May to 31 October from 2016 to 2022. Larval ticks, often found in large clusters, were removed with masking tape and counted. A subset from each cluster was collected and identified to genus [22].

There were very few observations to reconstruct a different site-level larval phenology for each year (mean 13.2 observations per site per year). Thus, the goal of this study was to explain differences in larval questing phenology across the sites not across the years. So larval questing data for each site were aggregated across the 7 years, and the average temperature at each site on each day across the 7 year time-period was estimated (effectively temperature normals from 2016 to 2022 for each site). PRISM (Parameter-elevation Regressions on Independent Slopes Model) modelled temperature was used to calculate these averages [23]. The PRISM takes weather station observations and then uses an elevation and slope informed model to interpolate daily weather across the continental United States at a resolution of 800 m.

PRISM-reported mean daily temperatures at each site from 2016 to 2022 were compiled [23]. For each Julian day, the average temperature over the 7 years was calculated, and these values were smoothed with a LOESS curve. PRISM gave above-canopy air temperatures which differ from that below the canopy [24]. Data from the National Ecological Observatory Network (NEON) were used to calculate this difference. The NEON measures a standard set of ecological and physical data at 80 sites across the United States. The two closest NEON sites were used: Bartlett Experimental Forest and Harvard Forest (150 and 183 km away from Middlebury, VT, the next closest NEON site is over 600 km away). For the two sites, soil-surface temperature on each day from 1 January 2016 to 31 December 2022 was compiled (NEON data product DP1.00005.001) [25]. The mean NEON-measured, below-canopy temperature each day was compared with the PRISM-modelled temperature, and a linear relationship was fit. This relationship was used to calculate the estimated below-canopy temperatures at my sites from PRISM reported above-canopy temperatures. See the electronic supplementary material for more information on this PRISM to below-canopy, field temperature adjustment.

### 2.3. Mechanistic phenology model

**Figure 2.**
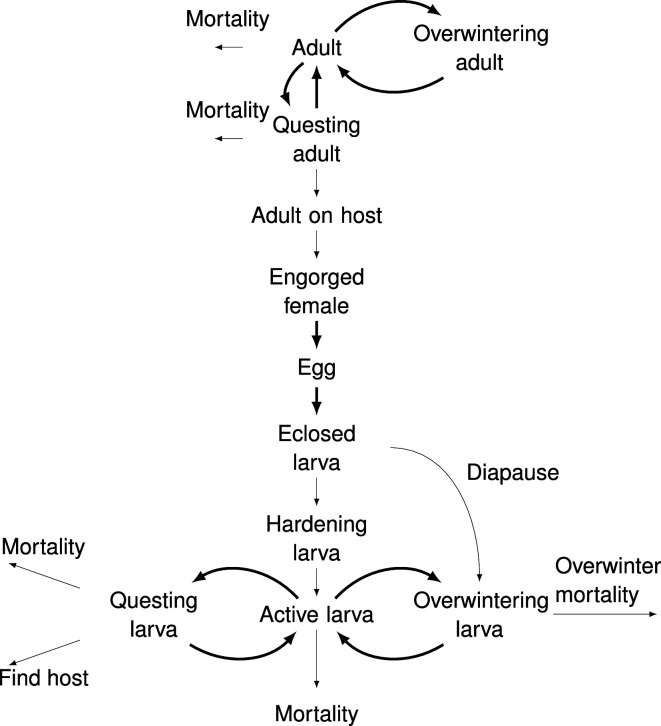
Flow diagram of the mechanistic larval questing phenology model. The model is made up of life-stage compartments and flows between those compartments. Bold arrows represent flow rates which are temperature-dependent. See table 1 for model parameters.

A mechanistic model to explain larval questing phenology was developed. This model was based on two by Ogden *et al*. [15,26], although with some modifications which are described below. It was made up of life-stage compartments and flows between them (figure 2). The flow between some compartments was temperature-dependent. The model tracked the daily fraction of ticks in each life-stage compartment. The model started with a cohort of active adults, whose questing was temperature-dependent. It assumes a piecewise linear function between temperature and the fraction of active adults questing [26]. This function increased from zero at a minimum questing temperature to one at a temperature of maximum questing and decreased symmetrically for higher temperatures. Although the function does not explicitly include the role of vapour pressure deficit (VPD), the decreasing questing at higher temperatures reflects how increased VPD at high temperatures decreases questing. Active ticks, questing or not, had a daily mortality rate, while questing ticks had a daily host-finding rate. The cohort of adult ticks started their activity in the late summer or fall when the temperature dropped below their maximum questing temperature. They continued questing until the temperature dropped below the minimum questing temperature, and the remaining active adult ticks entered the overwinter quiescence. They returned to activity once daily temperature passed the questing threshold the following spring. Thus, the model includes both fall- and spring-questing adults.

After an adult found a host, it spent a constant time period feeding and then dropped off engorged [27]. For these engorged adults, the days to oviposition were determined by thermal accumulation [28]. The threshold thermal accumulation varied between ticks with a normal distribution. The days to eclosion were also determined by thermal accumulation and varied between eggs [28]. The model assumed that a fraction of larvae eclosed after the summer solstice entered diapause and were not active until the following year [15]. The remaining fraction, and those eclosed before the summer solstice, experienced a period of hardening before they became active [29]. Active larval ticks followed the same rules as for adult ticks with a temperature-dependent fraction of larvae questing on each day. Active and questing larvae had a daily mortality rate, and questing larvae had a daily host-finding rate. The parameters for larvae differ from those for adults. Once the daily temperature dropped below the minimum threshold for larval questing, all remaining active larvae entered overwinter quiescence. A fraction of these larvae, and those which previously entered diapause, survived overwintering. These larvae then returned to activity once daily temperature passed the questing threshold the following spring. Values for each parameter were determined from the literature (table 1). A detailed description of the model can be found in the electronic supplementary material.

**Table 1.** Model parameters.

parameter	value	sources
min temp. for adult questing	3°C	[26]
temp. of max adult questing	8°C	[26]
host-finding rate for questing adults	0.04 d^-1^	[26]^a^
mortality rate for active and questing adults	0.006 d^-1^	[26]
time adults spend feeding on host	6 d	[27]
mean degree days (base 6°C) to oviposition	188.0°C	[28,30]
s.d. degree days (base 6°C) to oviposition	50.1°C	[28,30]
mean degree days (base 11°C) to eclosion	532.1°C	[28,30]
s.d. degree days (base 11°C) to eclosion	38.5°C	[28,30]
fraction of ticks eclosed after the summer solstice that enter overwinter diapause	0.5	[15]
number of days hardening (eclosion to activity)	7 days	[29]
min temp. for larval questing	10°C	[26,31]
temp. of max larval questing	25°C	[26,31]
host-finding rate for questing larvae	0.02 d^-1^	[26]^a^
mortality rate for active and questing larvae	0.01 d^-1^	[32,33]^b^
larval overwintering survival	0.45	[30,34–36]

^a^ Host-finding in [26] was host-density-dependent. The default rodent and deer densities from [26] were used to calculate the weekly host-finding rate. This weekly rate was converted to a daily rate given here.

^b^ In [32], larval mortality depends on RH. Here mortality was calculated based on 100% RH, because ticks spend most of their time in the leaf litter where the air is near saturated. [33] gives larval mortality of southern and northern ticks in southern and northern environmental conditions. This mortality value was calculated for northern ticks in northern conditions.

RH, relative humidity..

### 2.4. Model comparison

**Figure 3.**
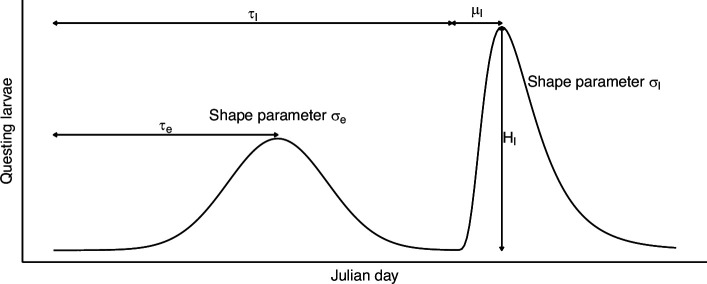
Phenomenological model of larval questing phenology from [37]. This model is composed of a shifted normal curve plus a lognormal curve. This phenomenologically recreates the early- and late-season larval questing peaks, respectively. The model has six parameters: $\tau_{e}$, the Julian day of peak early-season questing; $\sigma_{e}$, a shape parameter describing the spread around this peak; $\tau_{l}$, the Julian day of onset of late-season questing; $\mu_{l}$, the number of days between onset and peak late-season questing; $H_{l}$, the number of larvae questing at peak late-season activity; and $\sigma_{l}$, a shape parameter describing the spread around this peak. The height of the early-season peak is taken as one. It is not included as a free parameter since the curve will be later scaled to fit the density at each site. $H_{l}$ is included as it sets the height of the late-season peak relative to the early-season peak.

Four models were compared to see which best explained larval phenology (table 2). The mechanistic model is described above. This model was run on the below-canopy temperature at each site to get predicted larval phenologies at each site. A single mechanistic model with the average below-canopy temperature across all sites was also run. This represented a hypothesis that temperature explained phenology but temperature differences across the sites were not large enough to be important. A phenomenological model from Brunner & Ostfeld [37] was also included. This model used a shifted normal curve plus a shifted lognormal curve to give the bimodal form (early- and late-summer questing peaks) of larval phenology (figure 3). Here as well, this phenomenological model was fit to observed data at each site individually and fit to questing data averaged across all the sites. The four models were fit to the drag-cloth sampled larval questing data aggregated across the 7 years of the study. Models were fit using the mle2 function in the bbmle R package [38,39]. For all models, the purpose was to explain larval-questing phenology not the difference in larval density across sites, so in all cases, a free, site-level parameter scaled tick density. All models assumed a negative binomially distributed number of ticks with an additional site-level dispersion parameter. Models were compared with Akaike information criterion (AIC) [40].

**Table 2.** Models compared.

number	scale	type	hypothesis
1	site	mechanistic	the mechanistic model with elevational differences in temperature explains differences in phenology
2	mean	mechanistic	the mechanistic model with the average temperature across sites describes phenology at all sites; temperature differences across sites do not result in different phenology patterns
3	site	phenomenological	each site has its own bimodal phenology
4	mean	phenomenological	a single bimodal curve describes the phenology at all sites without elevational differences

The fraction of larvae questing in the early versus late summer is a key aspect of biological interest, since this determines what fraction of larvae quest synchronously (early summer) versus asynchronously (late summer) with nymphs. So, for the best-performing model, the observed versus predicted fraction of larvae questing in late summer was compared.

### 2.5. Data and code availability

Analyses were done in R with the bbmle, tidyverse, mgcv, cowplot and leaflet packages [38,39,41–43]. Data and relevant code for this research are stored at GitHub (https://github.com/dallenmidd/larval_phenology) and have been archived within the Zenodo repository (https://doi.org/10.5281/zenodo.14961346).

## 3. Results

Larvae per 200 m^2^ sample were overdispersed (median = 0, mean = 10.2, s.d. = 43.0, *n* = 1122). Larval ticks were not found at the second-to-highest elevation site, so it was omitted from the analysis leaving 12 sites. I assumed that all *Ixodes* larvae were *I. scapularis*. This is the only non-nidicolous *Ixodes* species in Vermont. Nidicolous ticks are rarely sampled with drag-cloth sampling. During this study, nymph and adult ticks were also collected and identified to species. 3933 *Ixodes* nymphs and 622 adults were found. Only three of these were not *I. scapularis*, one *I. muris* and two *I. cookei* nymphs. So, it is likely that almost all *Ixodes* larvae were *I. scapularis*. Gatewood *et al*. made the same assumption of drag-sampled *Ixodes* larvae in the eastern United States [12].

**Figure 4.**
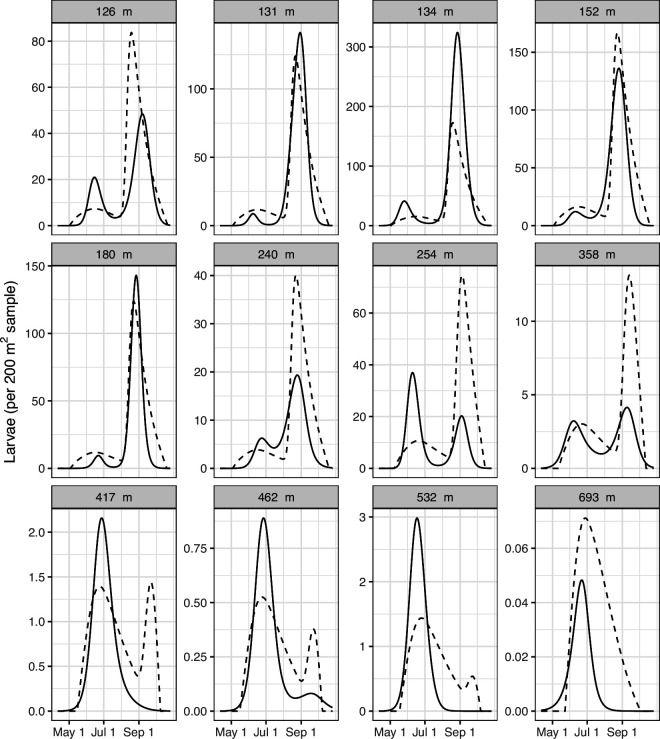
Larval phenology across the 12 sites where larvae were found. Sites labelled by their elevation above sea level in metres. Solid lines give a smoothed curve fit to the sampling data at each site aggregated across the 7 years of the study. The dotted lines give the predictions of the site-level, mechanistic model, which performed the best. The smoothed curves fit to sampling data are shown rather than raw data for clarity, but the models were fit to the raw sampling data.

Larval phenology followed a bimodal pattern with peaks in mid-June and late-August (figure 4). Larval density generally decreased with elevation (see y axes on figure 4); this same result for nymphal density was observed [21]. With an increase in elevation, the relative height of the early-summer peak increased while that of the late-summer peak decreased (figure 4).

**Figure 5.**
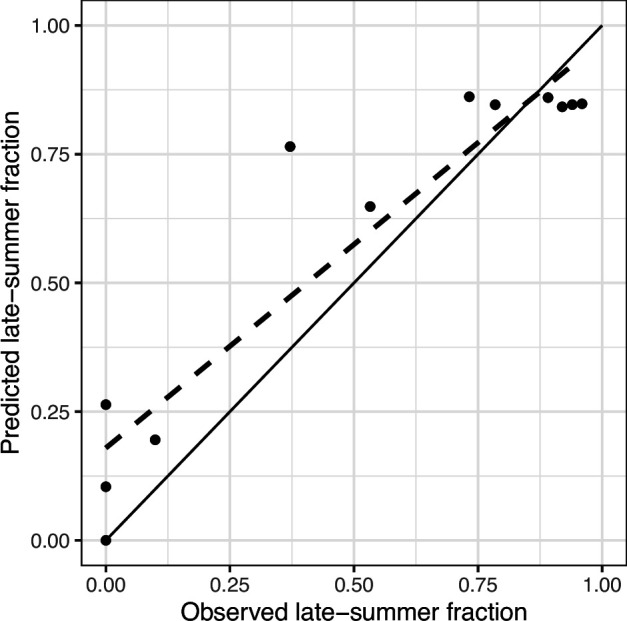
The observed and predicted fraction of larvae questing in the late summer. The prediction is based on the best-performing model, the site-level mechanistic one. The solid line gives the y=x line and the dotted line the trend line between observed versus predicted. The equation for the trend line is y=0.79x+0.18 with an $r^{2}=0.87$, so a good fit to the y=x line. The mean absolute error between observed and predicted was 0.12.

The result of the model comparison is shown in table 3. The phenomenological model fit to each site (model three) had the lowest negative log likelihood. This is expected as it fits a high-parameter function to each site individually. On the other hand, the site-level, temperature-driven mechanistic model had the lowest AIC. This model did a good job of reproducing the site-level differences in larval phenology with fewer free parameters. It reproduced the general pattern that with increasing elevation, the relative size of the late-summer peak decreased and early-summer peak increased (figure 4). At the highest elevation site, it correctly predicted that all larvae would be found in the early-summer peak. The model did a good job of explaining differences in the fraction of larvae questing in late summer across the sites (figure 5). Still, the model predictions had some key differences from the observed data: it predicted that the late-summer activity peak would be larger in mid- and high-elevation sites than observed and that the early-summer activity peak would be more spread out and persist for longer than it did.

**Table 3.** Model comparison: Each model had 24 parameters, density-scaling and dispersion parameters for each of the 12 sites. Those were the only parameters for the two mechanistic models because their phenologies were set by the mechanistic models which did not have any free parameters. The site-level phenomenological model had 72 additional parameters, the 6 parameters of the phenomenological model (figure 3) for each site. The mean-level phenomenological model had six additional parameters for the single phenomenological model.

number	model	parameters	NLL	ΔAIC
1	site mechanistic	24	1635	0
2	mean mechanistic	24	1655	20
3	site phenomenological	24+72=96	1578	87
4	mean phenomenological	24+6=30	1630	7

AIC, Akaike information criterion; NLL, negative log likelihood.

## 4. Discussion

Differences in larval tick questing phenology across an elevation gradient were found. With increased elevation, there was an increase in the fraction of early-summer versus late-summer questing ticks. A temperature-based larval questing phenology model was developed and parametrized from the literature. This model reproduced the observed pattern and performed better than three competing models.

Others have found differences in *Ixodes* larval phenology across much larger spatial scales [9,12,15] or across different years [17]. Here, dramatic differences over smaller spatial scales were found, presumably due to elevational differences in climate. These results are in line with the prediction that in cooler locations or years, the fraction of late-summer questing larvae decreases [12,17]. This is consistent with what is known of *I. scapularis* development. Eggs are laid by gravid females in early summer [34]. Then eclosion and questing are temperature-dependent [28]. So, more larvae are active in the summer when they are laid in warmer locations where they have faster development and warm late-summer temperature to quest. Otherwise, larvae overwinter and those observed in early summer are from the previous year’s cohort [34].

The temperature-dependent mechanistic model, which includes those processes, was able to replicate the observed pattern. Earlier attempts to explain variation in larval phenology across larger spatial scales found that differences in temperature-independent diapause explained that variation [15]. As such, they could not directly test how a single model, driven only by temperature, explained variation in larval phenology across sites. Variation in questing phenology at a small spatial scale, at which local adaptation should play a smaller role, was found, and a single mechanistic model explained this variation. This use of elevation over a small spatial scale is also a limitation of my approach. Although the model tested here works for a range of climates, it does so in a single geographic setting. And as Ogden *et al*. show, tick behaviour is different across its range [15]. So this model would need to be modified, likely by changing the temperature-independent diapause fraction parameter, to be applied in other geographic locations.

Though the model did a good job of describing larval phenology, there were clear differences between model predictions and observed larval questing. It predicted that early-summer larvae would continue questing longer into summer than observed. This could be because overwintered larvae have a higher mortality rate due to being near the end of their energy stores or experiencing greater desiccation risk as the summer progresses [33]. The model does not account for these as it includes a constant larval mortality rate.

The timing of larval feeding is important to the persistence of horizontally transmitted tick-borne pathogens. These pathogens are transmitted from one cohort of ticks to the next through vertebrate hosts. Pathogen persistence depends on the interaction of larval and nymphal questing synchrony with the duration of infectiousness of the pathogen in the vertebrate host [6,7]. In the northeastern United States, nymphal *I. scapularis* ticks quest from late-spring to early-summer. Thus, at the low elevation sites, there is predominantly asynchronous larval and nymphal questing, while at high elevation ones, it is predominantly synchronous. This could result in selection for long-duration infectious strains of *B. burgdorferi* at low elevations [12].

Ultimately, we want to understand how climate affects the transmission and persistence of tick-borne diseases. This transmission is the result of a number of biological processes, each with its own climate-dependence. To accurately map, predict and understand how climate change will influence tick-borne diseases, we need a mechanistic understanding of how climate affects each of these processes [44]. These can then be incorporated into tick population models [26,45]. These models can be projected forward to see how climate change should influence future populations of ticks or tick-borne pathogens [46–48]. But these projections will only work if the underlying models have the correct climate dependence on each biological process. This study provides such a validation by showing mechanistically how a key biological process, larval phenology, is affected by temperature.

## Data Availability

Data and relevant code for this research are stored at GitHub [49] and have been archived within the Zenodo repository [50]. Supplementary material is available online [51].
